# Supplementary material for: Nuclear Export Inhibitor KPT-8602 Synergizes with PARP Inhibitors in Escalating Apoptosis in Castration Resistant Cancer Cells
Source: Int J Mol Sci. 2021 Jun 22;22(13):6676. doi: 10.3390/ijms22136676 (PMC8268282; doi:10.3390/ijms22136676)
Supplement: Supplementary file 1 [file ijms-22-06676-s001.zip › ijms-1260576-supplementary.pdf]

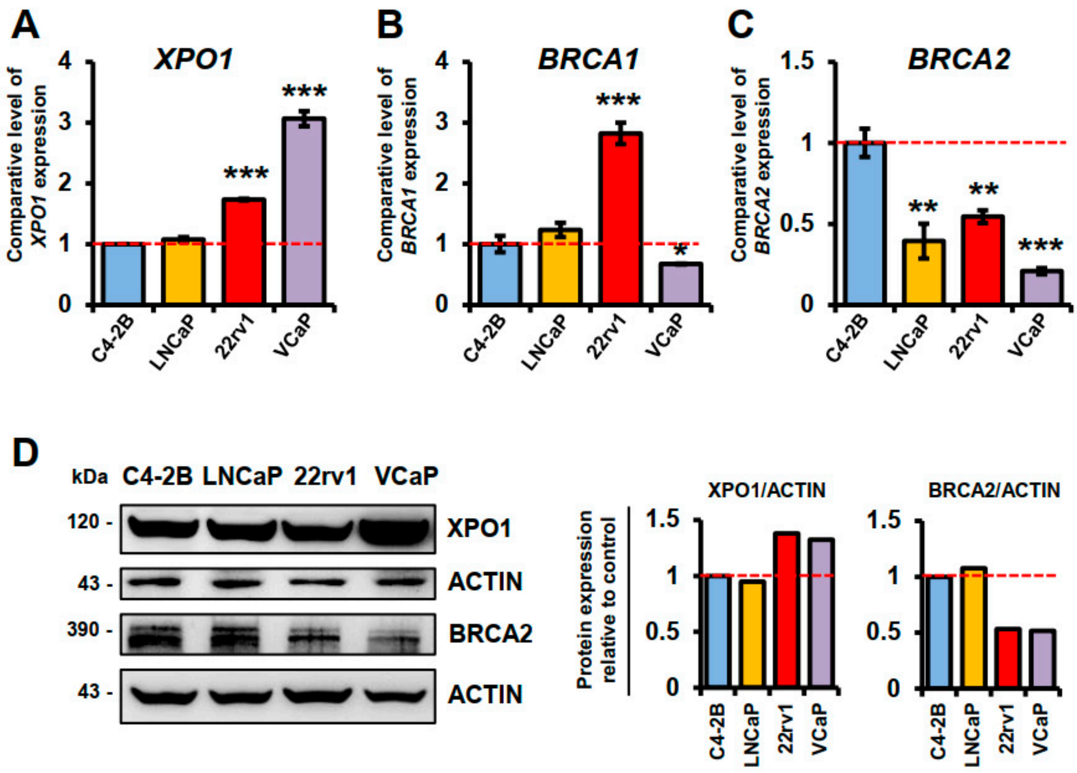

Figure S1. Expression of XPO1, BRCA1, BRCA2 in a panel of prostate cancer cells. A-C. Total RNA was extracted from growing culture at about 80% confluency. Gene expression was determined by real-time RT-qPCR. D. Protein lysate was obtained from a culture growing at about 80% confluency. Basal expression of XPO1 and BRCA2 proteins were determined by western blotting technique. Side panel shows relative protein expression. Densitometry analysis was performed by utilizing NIH ImageJ 1.5Oi software.

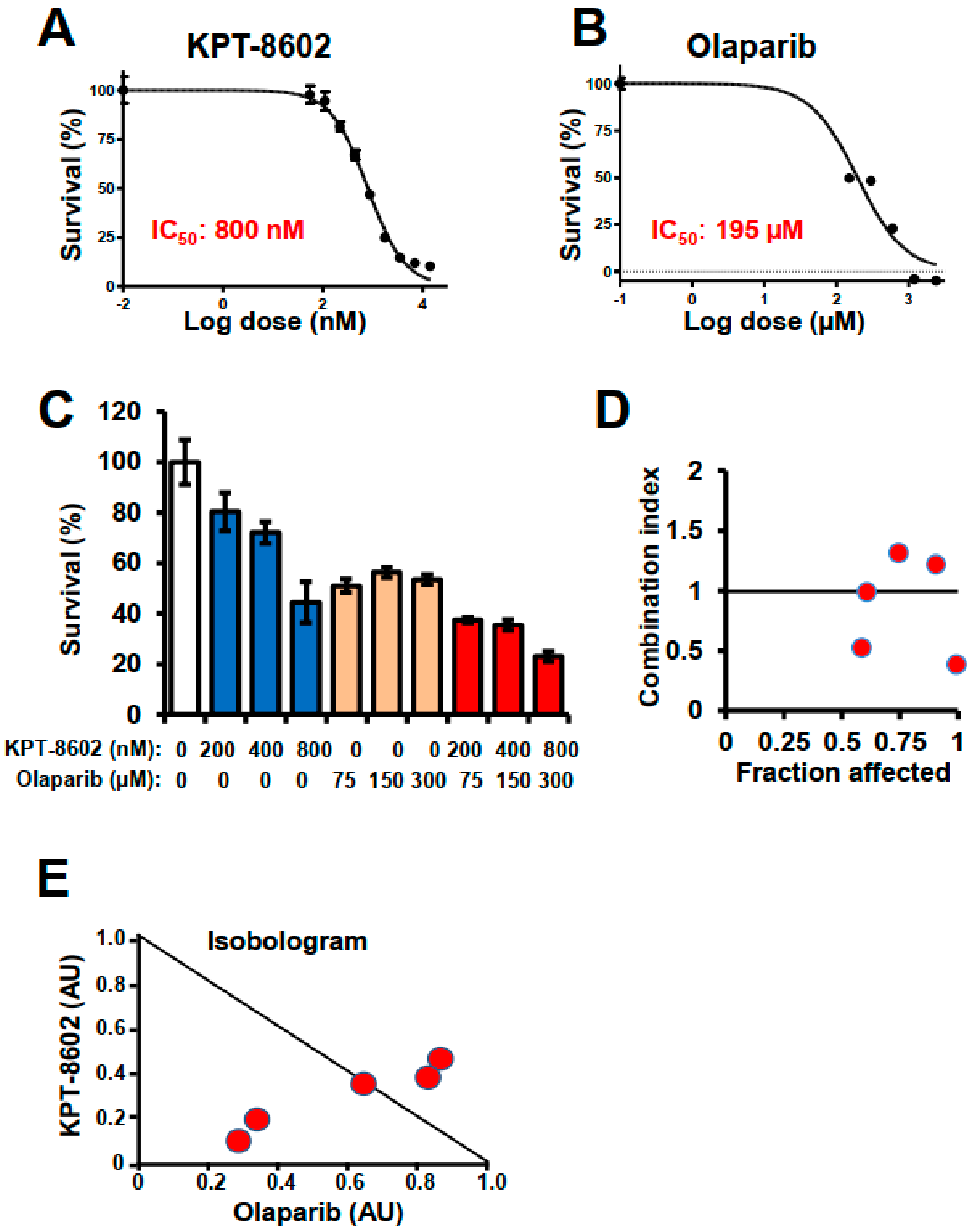

Figure S2. KPT-8602 synergizes with PARP inhibitor olaparib in C4-2B prostate cancer cells. A-B. Dose response curves for single agents. C4-2B cells were seeded at 4000 cells per well and treated on the next day with 62.5 nM - 5000 nM doses of KPT-8602 or 125 µM - 2000 µM doses of olaparib for 72 hours. MTT assay was performed to determine the growth inhibition. C. Selected dose combinations. Three doses of KPT-8602 and olaparib between 200 nM - 800 nM and 75 µM - 300 µM respectively were used both alone and in combination to determine the synergistic effect.D. Combination Index and E. Normalized isobologram were generated using Calcusyn 2.1 software.

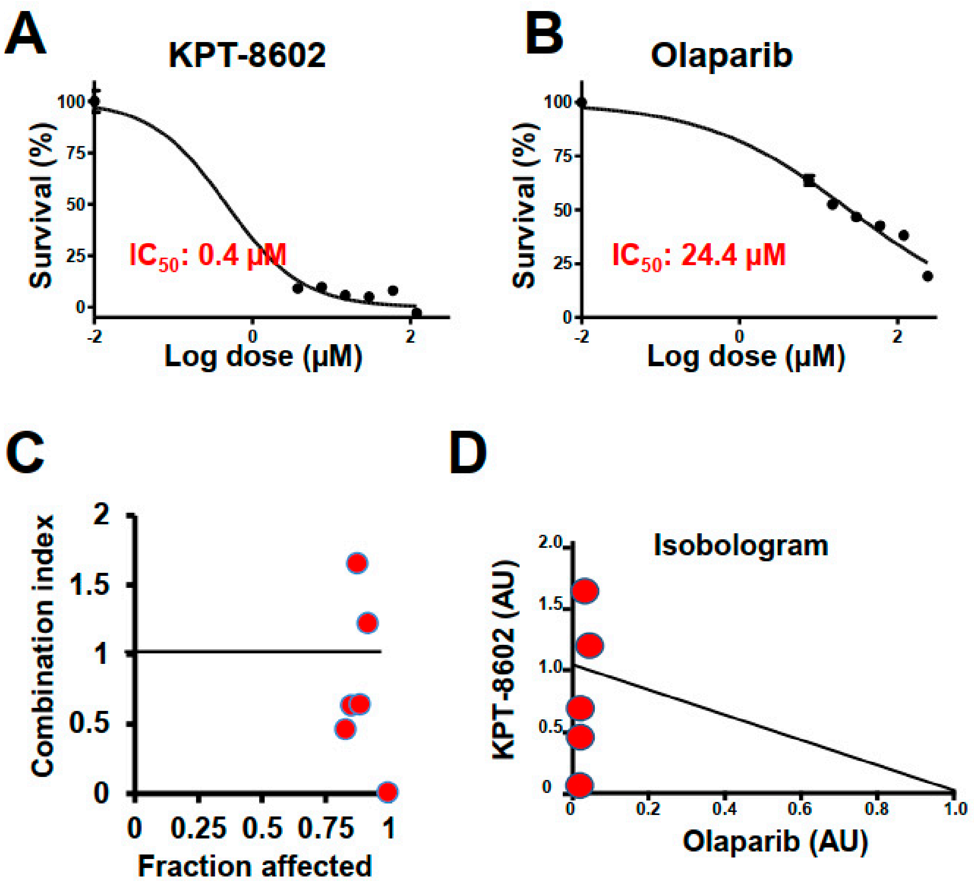

Figure S3. KPT-8602 synergizes with PARP inhibitor olaparib in LNCaP prostate cancer cells. A-B. Dose response curves for single agents. LNCaP cells were seeded at 4000 cells per well and treated on the next day with 3.75 - 120 µM doses of KPT-8602 or 7.5 - 240 µM doses of olaparib for 72 hours. MTT assay was performed to determine the growth inhibition. C. Combination Index and D. Normalized isobologram were generated using Calcusyn 2.1 software. Six doses of KPT-8602 and olaparib between 3.75 µM -120 µM and 75 µM - 300 µM respectively were used both alone and in combination to determine the synergistic effect.

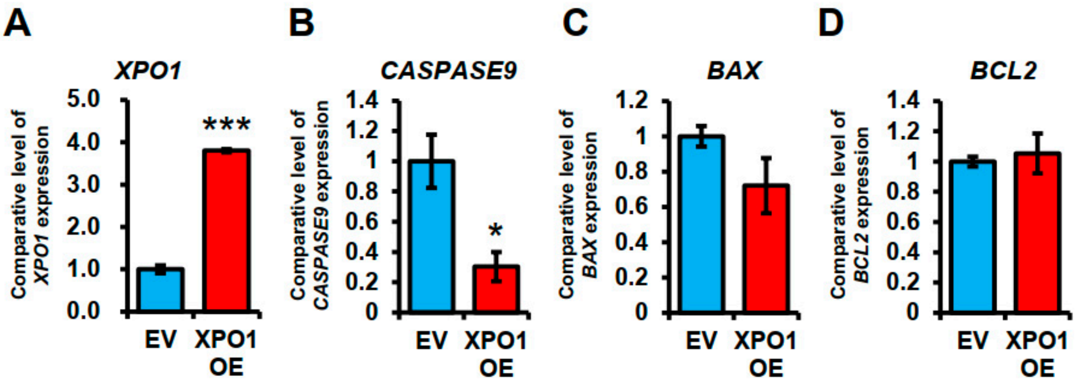

Figure S4. Expression of apoptosis related genes in XPO1 overexpressed SNU-1 cancer cells. A-D. Total RNA was extracted from SNU-1 cells after 48 hrs of transient transfection. The *XPO1, CASPASE 9*, *BAX* and *BCL2* gene expressions were determined by SYBR green real-time RT-qPCR. Experiments have been done in triplicates. Error bars represent standard deviation. * $p < 0.05$; *** $p < 0.001$. EV, empty vector; OE, over expression.

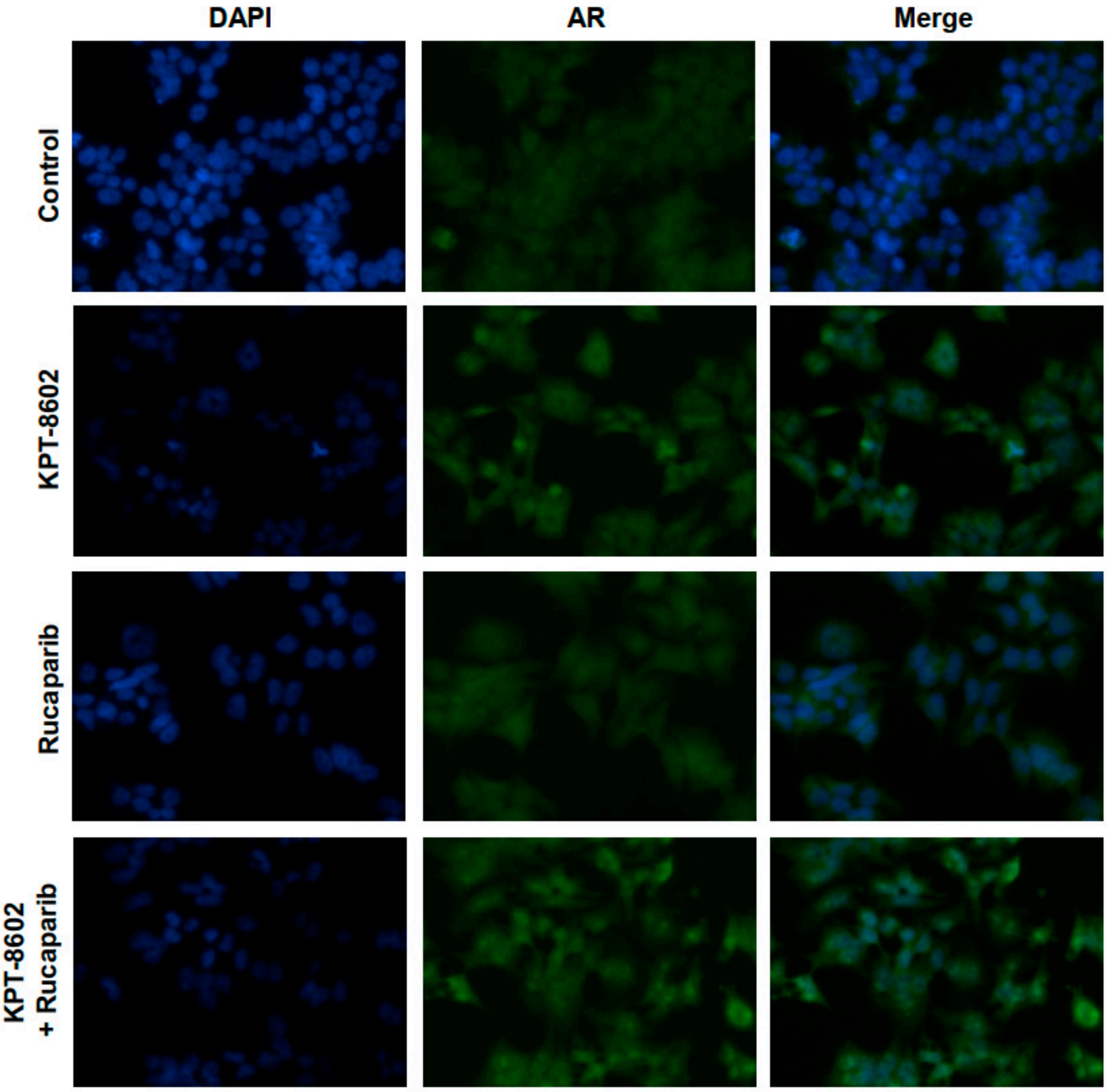

Figure S5. Immunofluorescence (IF) staining of AR in HEK293 cells. Cells were grown in poly-L-lysine coated chamber slides and treated with 2 μM dose of KPT-8602 and 10 μM dose of rucaparib alone or in combination. After 24 hrs cells were fixed, permeabilized and stained with 1:600 dilution of AR (green) antibody. Nuclei were stained with DAPI (blue).

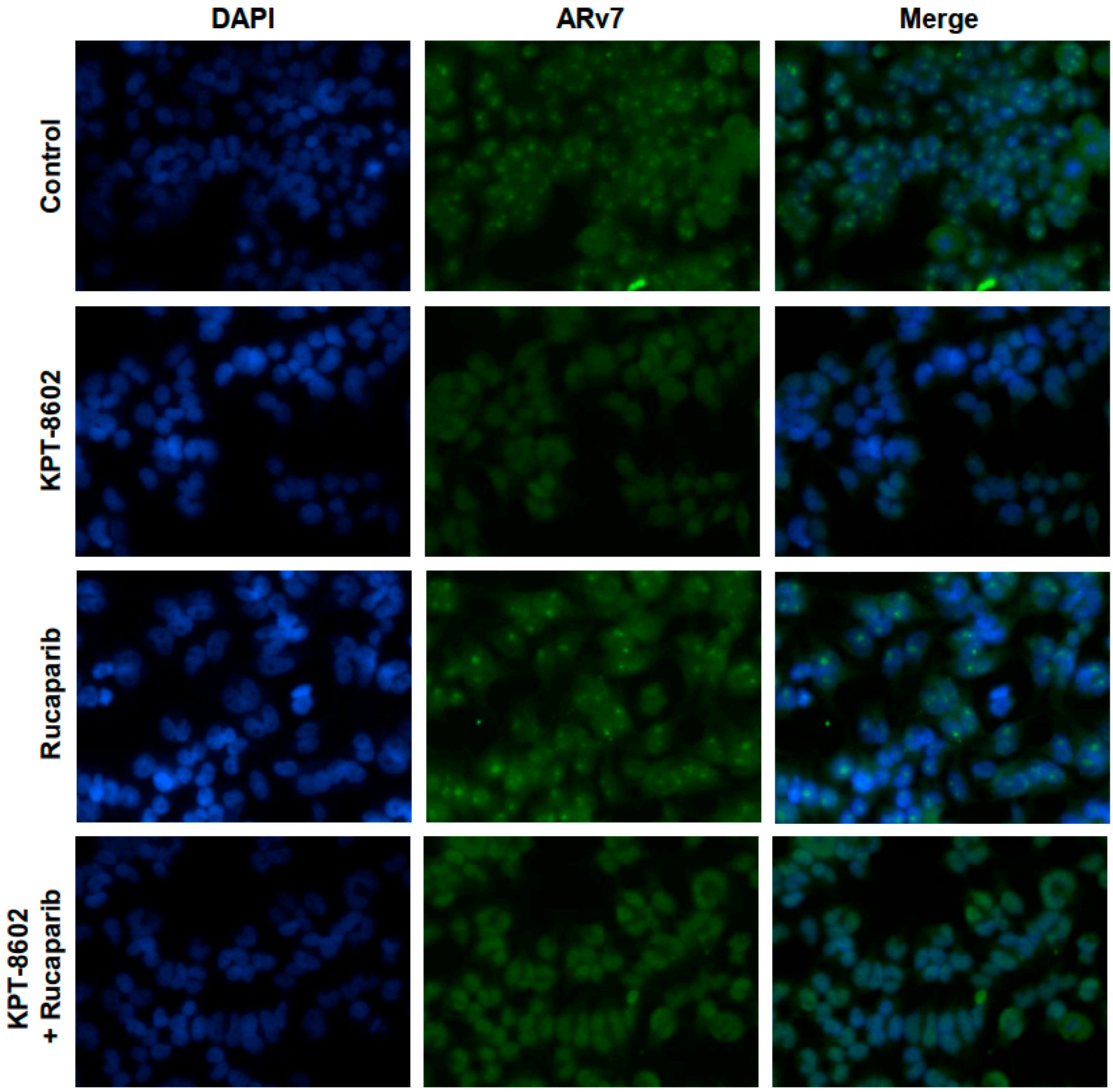

Figure S6. Immunofluorescence (IF) staining of ARv7 in HEK293 cells. Cells were grown in poly-L-lysine coated chamber slides and treated with 2 µM dose of KPT-8602 and 10 µM dose of rucaparib alone or in combination. After 24 hrs cells were fixed, permeabilized and stained with 1:100 dilution of ARv7 (green) antibody. Nuclei were stained with DAPI (blue).

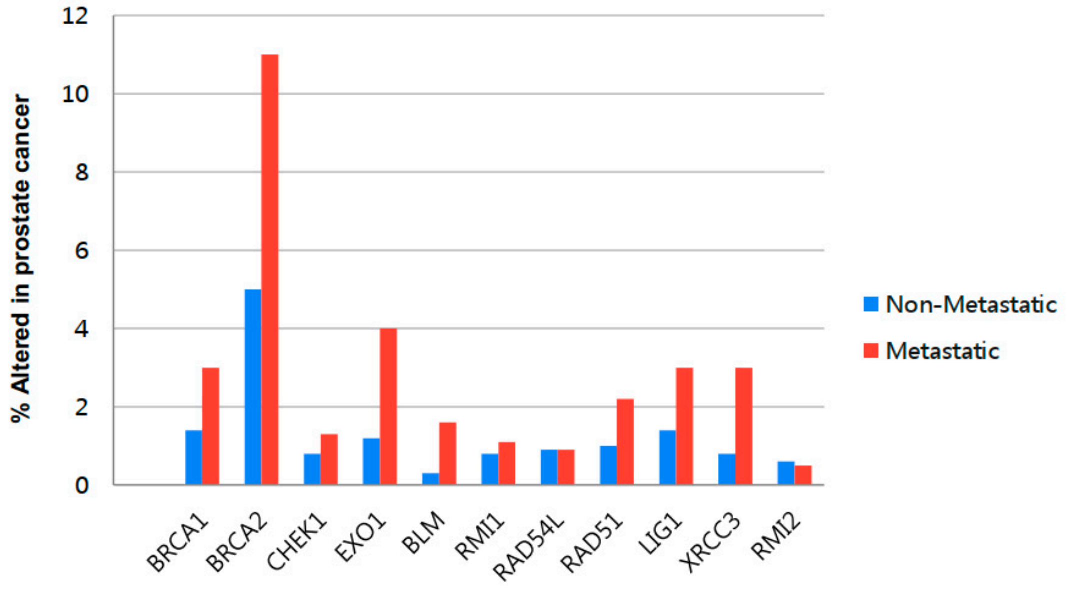

Figure S7. Alteration of DNA damage markers in metastatic and non-metastatic prostate adenocarcinoma in TCGA database. Alterations in *BRCA1, BRCA2, CHEK1, EXO1, BLM, RMI1, RAD54L, RAD51, LIG1, XRCC3* and *RMI2* genes were compared in prostate cancer datasets available at cBioPortal for Cancer Genomics (http://www.cbioportal.org). Alterations include gene amplification, deep deletion, different types of mutation and fusion of genes.

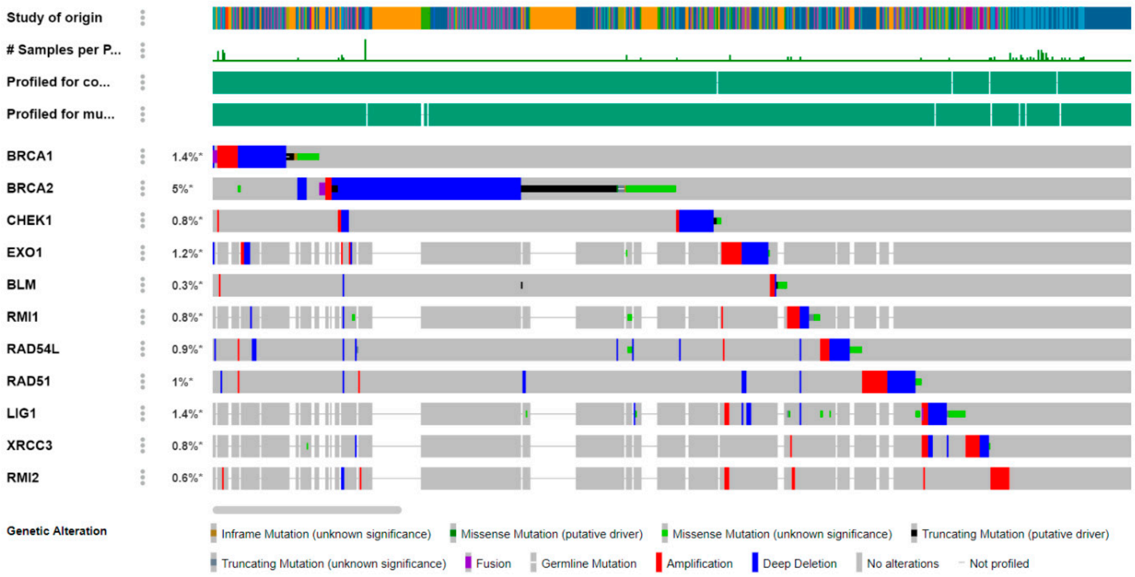

Figure S8. OncoPrint image of DNA damage markers in non-metastatic prostate adenocarcinoma in TCGA database. Alterations in *BRCA1, BRCA2, CHEK1, EXO1, BLM, RMI1, RAD54L, RAD51, LIG1, XRCC3* and *RMI2* genes has presented for in non-metastatic prostate cancer datasets available at

cBioPortal for Cancer Genomics (http://www.cbioportal.org). Alterations are color coded as shown at the bottom of the figure which includes gene amplification, deep deletion, different types of mutation including driver mutations and mutation of unknown significance and fusion of genes.

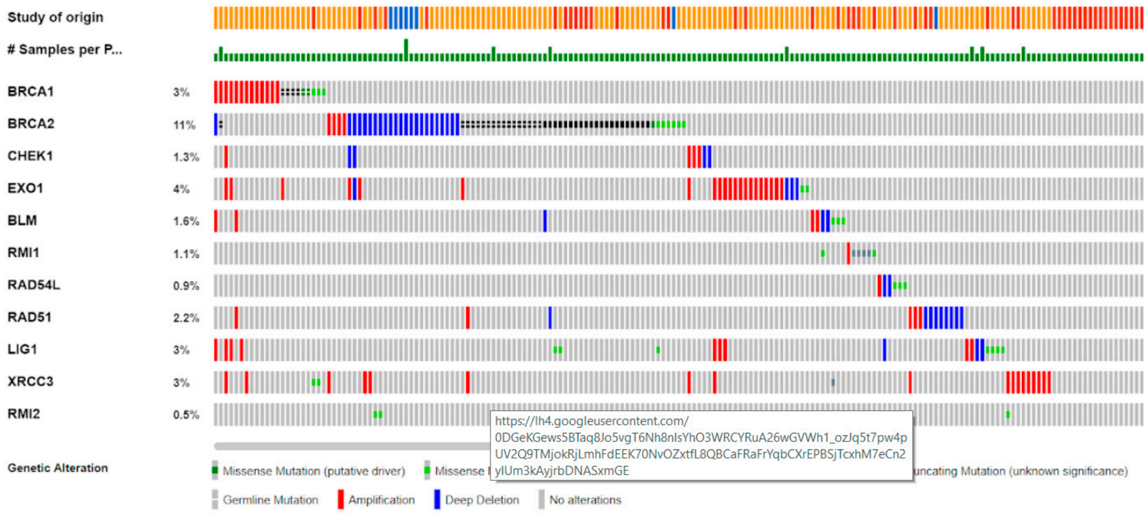

Figure S9. OncoPrint image of DNA damage markers in metastatic prostate adenocarcinoma in TCGA database. Alterations in *BRCA1*, *BRCA2*, *CHEK1*, *EXO1*, *BLM*, *RMI1*, *RAD54L*, *RAD51*, *LIG1*, *XRCC3* and *RMI2* genes has presented for in metastatic prostate cancer datasets available at cBioPortal for Cancer Genomics (http://www.cbioportal.org). Alterations are color coded as shown at the bottom of the figure which includes gene amplification, deep deletion and different types of mutation including driver mutations and mutation of unknown significance.

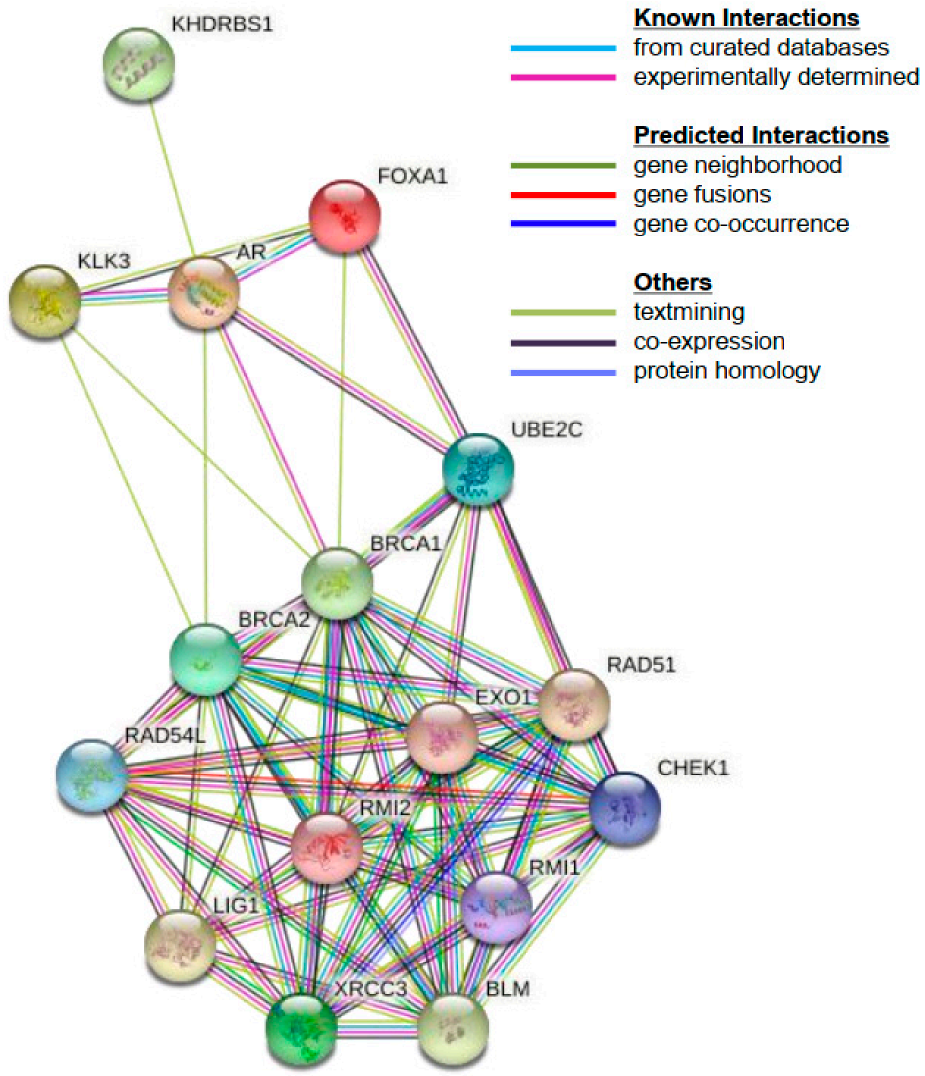

Figure S10. Proteomic interaction between androgen signaling and DNA damage repair molecules as determined by STRING analysis (https://string-db.org; version: 11.0). The nodes in the network represent proteins. The filled nodes indicate some 3D structure is known or predicted. A line connection between proteins represents protein-protein interaction. The color codes of line are depicted in the figure. The associations are meant to be specific and meaningful with associated proteins jointly contributing to a shared function. This does not necessarily mean that the proteins are physically binding each other.

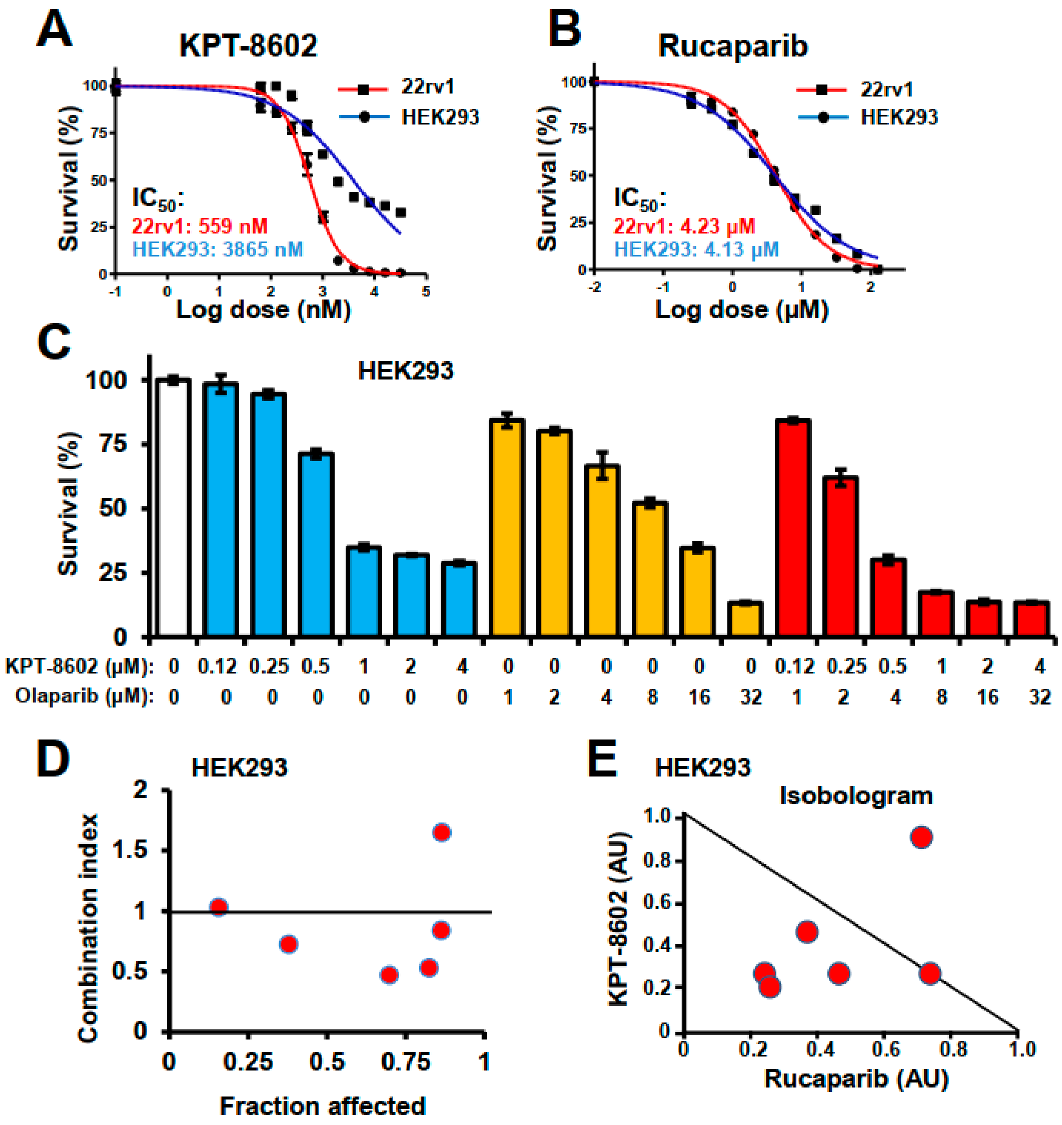

Figure S11. Drug response and synergy between KPT-8602 and rucaparib in non-cancerous cells. A-B. 22rv1 and HEK293 cells were treated with varying doses of KPT-8602 or rucaparib for 72 hours. MTT assay was performed to determine the growth inhibition and represented as the dose response curve. C. The growth inhibition bar diagram for HEK293 with single or combinations of KPT-8602 and rucaparib with selected doses indicated in the figure. D-E. The combination indexes and isobolograms of figure C were generated using Calcusyn 2.1 software. All experiments have been done in triplicates. Error bar represents standard deviation. The combination index (CI) values are shown in Table S2.

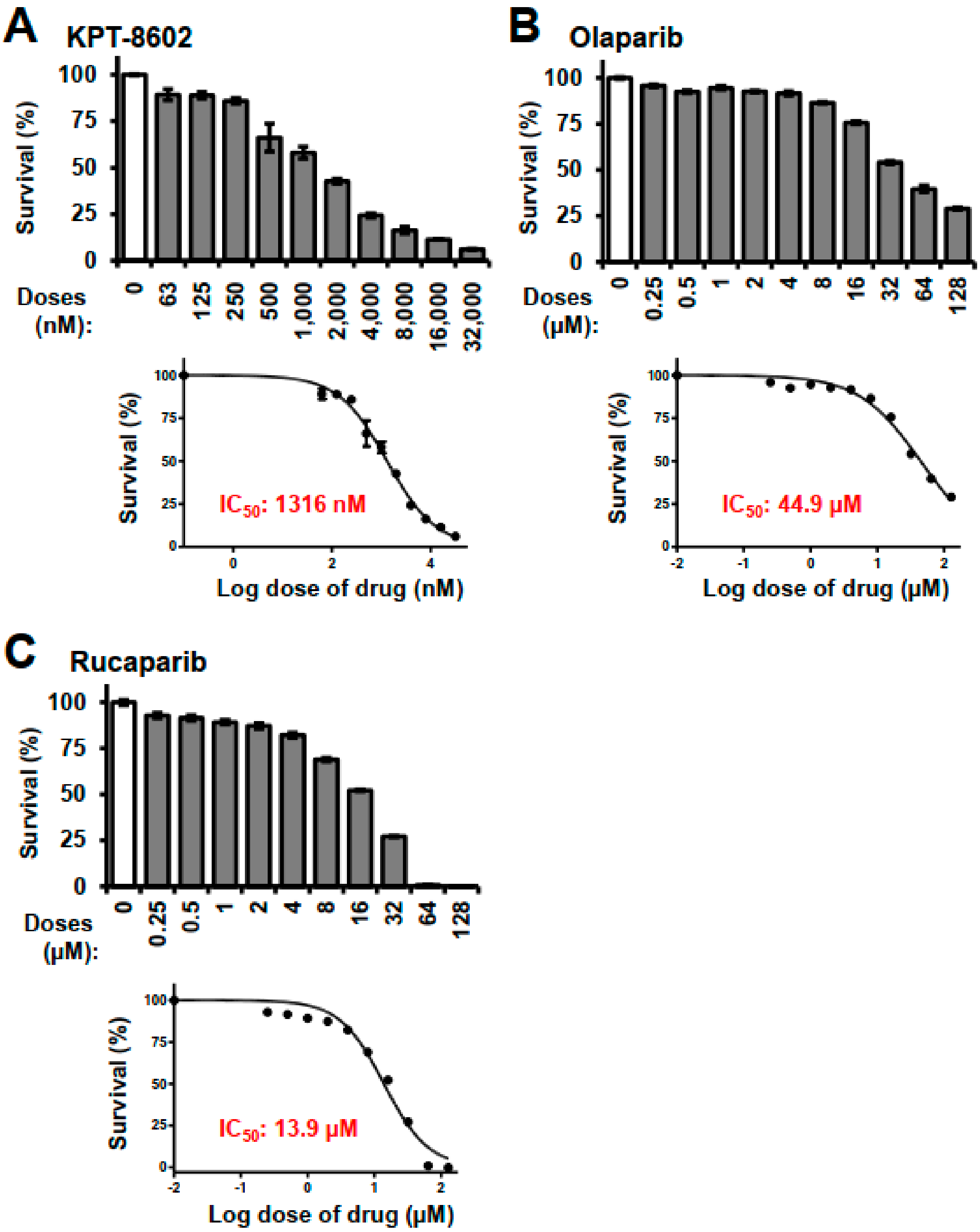

Figure S12. Drug sensitivity of normal human pancreatic islet cells against KPT-8602 and PARPi. A-C. The normal pancreatic islet cells were treated with varying doses of KPT-8602, olaparib or rucaparib for 72 hours. MTT assay was performed to determine the growth inhibition and represented as histogram and dose response curve. The IC_{50} values for all drugs were calculated using GraphPad Prism software. All experiments have been done in triplicates. Error bar represents standard deviation.

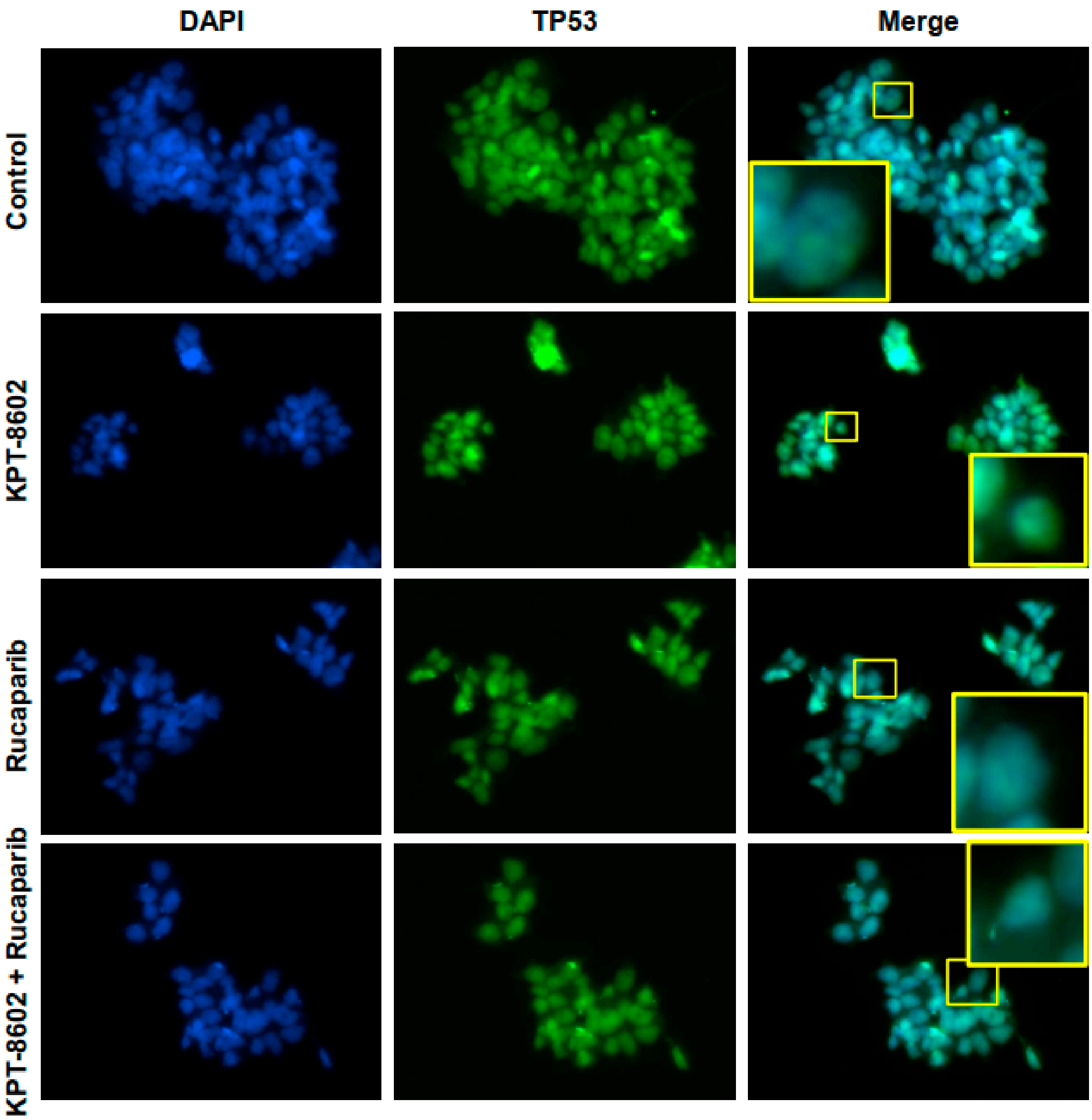

Figure S13. Immunofluorescence (IF) staining of TP53 in 22rv1 cells. Cells were grown in cell culture treated chamber slides and treated with 2 μM dose of KPT-8602 and 10 μM dose of rucaparib alone or in combination. After 4 hrs, cells were fixed, permeabilized and stained with 1:2000 dilution of TP53 (green) antibody. Nuclei were stained with DAPI (blue).

Table S1. Alteration of DNA damage genetic markers in metastatic and non-metastatic prostate adenocarcinoma in TCGA database.

DNA damage markers (genes)	Non-Metastatic (alteration %)	Metastatic (alteration %)	Fold higher in metastatic prostate cancer
BRCA1	1.4	3	2.1
BRCA2	5	11	2.2
CHEK1	0.8	1.3	1.6
EXO1	1.2	4	3.3
BLM	0.3	1.6	5.3
RMI1	0.8	1.1	1.4
RAD54L	0.9	0.9	1.0
RAD51	1	2.2	2.2
LIG1	1.4	3	2.1
XRCC3	0.8	3	3.8
RMI2	0.6	0.5	0.8

Table S2. Combination index (CI) values for KPT-8602 and rucaparib co-treatment in HEK293 cells.

KPT-8602 (nM)	Rucaparib (μM)	Fraction affected	CI value
125	1	0.157173	1.03
250	2	0.379538	0.724
500	4	0.699048	0.469
1000	8	0.825077	0.527
2000	16	0.862675	0.839
4000	32	0.865257	1.648

CI<1 indicates synergistic effect, CI=1, indicates additive effect and CI>1, indicates antagonistic effect.
